# Health Tract, No. 171

**Published:** 1863-09

**Authors:** 


### HEALTH TRACT, No. 171.

### GREAT EATERS

Never live long. A voracious appetite, so far from being a sign of health, is a certain indication of disease. Some dyspeptics are always hungry ; feel best when they are eating, but as soon as they have eaten they enter torments, so distressing in their nature, as to make the unhappy victim wish for death. The appetite of health is that which inclines moderately to eat, when eating time comes, and which, when satisfied, leaves no unpleasant reminders. Multitudes measure their health by the amount they can eat ; and of any ten persons, nine are gratified at an increase of weight, as if mere bulk were an index of health ; when, in reality, any excess of fatness is, in proportion, decisive proof of existing disease ; showing that the absorbents of the system are too weak to discharge their duty ; and the tendency to fatness, to obesity, increases, until existence is a burden, and sudden death closes the history. Particular inquiry will almost unvaryingly elicit the fact, that a fat person, however rubicund and jolly, is never well, and yet they are envied.

While great eaters never live to an old age, and are never for a single day without some "symptom," some feeling sufficiently disagreeable to attract the mind's attention unpleasantly, small eaters, those who eat regularly of plain food, usually have no "spare flesh," are wiry and enduring, and live to an active old age. Remarkable exemplifications of these statements are found in the lives of centenarians of a past age. Galen, one of the most distinguished physicians among the ancients, lived very sparingly after the age of twenty-eight, and died in his hundred and fortieth year. Ketigern, who never tasted spirit or wine, and worked hard all his life, reached a hundred and eighty-five years. Jenkins, a poor Yorkshire fisherman, who lived on the coarsest diet, was one hundred and sixty-nine years old when he died. Old Parr lived to a hundred and fifty-three ; his diet being milk, cheese, whey, small beer, and coarse bread. The favorite diet of Henry Francisco, who lived to one hundred and forty, was tea, bread and butter, and baked apples. Ephraim Pratt, of Shutesbury, Massachusetts, who died aged one hundred and seventeen, lived chiefly on milk, and even that in small quantity ; his son Michael, by similar means, lived to be a hundred and three years old. Father Cull, a Methodist clergyman, died last year at the age of a hundred and five, the main diet of his life having been salted swine's flesh (bacon) and bread made of Indian meal. From these statements, nine general readers out of ten will jump to the conclusion that milk is "healthy," as are baked apples and bacon. These conclusions do not legitimately follow. The only inference that can be safely drawn is from the only fact running through all these cases—that plain food and a life of steady labor tend to a great age. As to the healthfulness and life-protracting qualities of any article of diet named, nothing can be inferred, for no two of the men lived on the same kind of food ; all that can be rationally and safely said is, either that they lived so long in spite of the quality of the food they ate, or that their instinct called for a particular kind of food ; and the gratification of that instinct instead of its perversion, with a life of steady labor, directly caused healthfulness and great length of days. We must not expect to live long by doing *any one thing* which an old man did, and omit all others, but by doing *all* he did, that is, work steadily, as well as eat mainly a particular dish.

